# Physical, Psychological, and Social Factors Associated with Exacerbation-Related Hospitalization in Patients with COPD

**DOI:** 10.3390/jcm9030636

**Published:** 2020-02-27

**Authors:** Mieke R.C. Crutsen, Spencer J. Keene, Daisy J.A. Janssen, Nienke Nakken, Miriam T. Groenen, Sander M.J. van Kuijk, Frits M.E. Franssen, Emiel F.M. Wouters, Martijn A. Spruit

**Affiliations:** 1 Department of Pulmonary Functioning, Maastricht University Medical Centre (MUMC+), 6229 HX Maastricht, The Netherlands; 2 Department of Research and Development, CIRO+, 6058 NM Horn, The Netherlandsmartijnspruit@ciro-horn.nl (M.A.S.); 3 Institute of Applied Health Research, College of Medical and Dental Sciences, University of Birmingham, Birmingham B15 2TT, UK; 4 Department of Clinical Pharmacy and Toxicology, Maastricht University Medical Centre+, 6229 HX Maastricht, The Netherlands; 5 Department of Health Services Research, CAPHRI, Maastricht University, 6229 ER Maastricht, The Netherlands; 6 Department of Clinical Epidemiology and Medical Technology Assessment, Maastricht University Medical Centre (MUMC+), 6229 HX Maastricht, The Netherlands; 7 Department of Respiratory Medicine, Maastricht University Medical Centre (MUMC+), 6229 HX Maastricht, The Netherlands; 8 NUTRIM School of Nutrition and Translational Research in Metabolism, Maastricht University Medical Centre (MUMC+), 6229 HX Maastricht, The Netherlands; 9 REVAL-Rehabilitation Research Center, BIOMED-Biomedical Research Institute, Faculty of Medicine and Life Sciences, Hasselt University, 3500 Diepenbeek, Belgium

**Keywords:** chronic obstructive pulmonary disease, hospitalization, disease progression, prognosis, predictors

## Abstract

Background and objective: Exacerbation(s) of chronic obstructive pulmonary disease (eCOPD) entail important events describing an acute deterioration of respiratory symptoms. Changes in medication and/or hospitalization are needed to gain control over the event. However, an exacerbation leading to hospitalization is associated with a worse prognosis for the patient. The objective of this study is to explore factors that could predict the probability of an eCOPD-related hospitalization. Methods: Data from 128 patients with COPD included in a prospective, longitudinal study were used. At baseline, physical, emotional, and social status of the patients were assessed. Moreover, hospital admission during a one year follow-up was captured. Different models were made based on univariate analysis, literature, and practice. These models were combined to come to one final overall prediction model. Results: During follow-up, 31 (24.2%) participants were admitted for eCOPD. The overall model contained six significant variables: currently smoking (OR = 3.93), forced vital capacity (FVC; OR = 0.97), timed-up-and-go time (TUG-time) (OR = 14.16), knowledge (COPD knowledge questionnaire, percentage correctly answered questions (CIROPD_%correct_)) (<60% (OR = 1.00); 60%–75%: (OR = 0.30); >75%: (OR = 1.94), eCOPD history (OR = 9.98), and care dependency scale (CDS) total score (OR = 1.12). This model was well calibrated (goodness-of-fit test: *p* = 0.91) and correctly classified 79.7% of the patients. Conclusion: A combination of TUG-time, eCOPD-related admission(s) prior to baseline, currently smoking, FVC, CDS total score, and CIROPD_%correct_ allows clinicians to predict the probability of an eCOPD-related hospitalization.

## 1. Introduction

Exacerbation(s) of chronic obstructive pulmonary disease (eCOPD) are important events that are defined as a deterioration of a patient’s stable condition beyond normal day-to-day variations and are acute in onset [1]. Changes in medication and/or hospitalization are needed to gain control over the event. Dyspnoea, productive cough, sputum purulence, increased sputum volume, and fever are the most distinct symptoms of eCOPD [2]. However, patients may also present themselves with more non-specific symptoms such as worsening exercise tolerance, fluid retention, increased fatigue, insomnia or sleepiness, depression, acute confusion, and/or general malaise [3,4]. 

An eCOPD is not only an important clinical endpoint in patients with COPD, but it is also a risk factor within itself for additional adverse outcomes. Patients suffering from severe eCOPD often require admission to a hospital, which is associated with a poor prognosis for the patient [5,6]. Suissa and colleagues reported in 2012 that 50% of the population died within 3.6 years after their first severe eCOPD [7]. A more recent study by Van Hirtum and colleagues reported a 5 year survival of 43.7% following an eCOPD-related hospitalization [8]. The prognosis of patients hospitalized for eCOPD changed positively over the years [9]. However, despite improved therapies and the emphasis on early recognition of exacerbation, the prevalence and the burden of severe exacerbations remain high. eCOPD leads to an accelerated decline in lung function and health status and to an increased likelihood of mortality [10]. A significant percentage of patients will be re-hospitalized during the first month after their initial discharge [6,10]. Hospital readmission for eCOPD is a major problem, since it reinforces the burden of the disease [11,12]. Hospitalization due to eCOPD is not only related to an increased risk for hospital readmission and mortality, but it is also associated with an increased risk for cardiovascular events [13]. 

Several studies identified risk factors associated with an increased likelihood of frequent prospective eCOPD. The strongest association found in the ECLIPSE study is a prior history of exacerbations [14]. Other risk factors that could predict an increased risk of future eCOPD-related hospitalizations are an impaired FEV_1_ [15,16,17,18], increased degree of dyspnoea [15,19], comorbidities such as diabetes mellitus, cardiac insufficiency, or ischemic heart disease [17,20], poor health status [15], older age [21], weight loss during hospitalization and/or low BMI [22], current smoking [16,23], long-term oxygen use [15], and low socioeconomic status [20,24]. A systematic review and meta-analysis by Atlantis and colleagues [25] provided evidence that depression and anxiety are also associated with an increased risk of eCOPD and possible disease-related mortality [25]. Besides physical and psychological factors, social factors might play “an equally important role” in predicting eCOPD [20]. For example, Janssen and colleagues showed that patients with advanced chronic organ failure (including COPD) who have an increased degree of care dependency were at increased risk of dying; however, they did not investigate the risk of eCOPD [26]. 

Identifying predictors of an eCOPD-related hospitalization could help to reduce the number of eCOPD through early recognition and possible treatment of these factors. This seems a crucial step in optimizing patient management decisions to reduce the risk of prospective severe exacerbations. In this study, we aimed to investigate physical, psychological, and social factors associated with an increased risk for eCOPD-related hospitalization in patients with moderate to very severe COPD. 

## 2. Materials and Methods

### 2.1. Study Design

The current study used data from the “Home Sweet Home” study [27], a prospective, longitudinal observational study on the home environment of patients with COPD Global Initiative for Chronic Obstructive Lung Disease classification (GOLD-class) II, III, or IV. The study population consisted of patients who were recruited by their chest physician or a respiratory nurse during hospital admission or at the outpatient clinic in four hospitals throughout the south-eastern part of the Netherlands. In addition, participants participating in the study “Correlates of CAT” (NTR3416) who met the inclusion criteria and were willing to participate in further research were additionally asked to participate. Informed consent was obtained at the start of the first visit. Data collection took place from July 2013 until April 2016 according to the study protocol [27]. The ethics approval of the “Home Sweet Home” study has been obtained from the Medical Ethical Committee of the Catharina Hospital Eindhoven, The Netherlands (NL42721.060.12/M12-1280) and is registered in the Dutch trial register (NTR3941). Current study is not involving human subjects, as data is used from the “Home Sweet Home” study. Therefore, the Medical Research Involving Human Subjects Act (WMO) does not apply to this study. Official approval was not required by the Institutional review board: Medical Ethics Review Committee of azM/UM Maastricht, the Netherlands.

### 2.2. Study Population

A total of 194 participants with COPD were enrolled and followed for 12 months. Data were collected during home visits at baseline and follow-up. Patients were eligible if they: (1) had moderate to severe COPD (GOLD-class II, III, or IV) [28]; (2) had no eCOPD <4 weeks before enrollment; (3) were able to speak and/or understand Dutch; and (4) provided written informed consent. Participants were excluded if they were unable to complete study questionnaires because of cognitive impairment (a score of ≥10 on the Short Blessed Test [29]) and if no outcome was reported during follow-up. 

### 2.3. Outcome and Predictors

Online Supplementary Materials Table S1 shows variables measured in the “Home Sweet Home” study categorized according to their purpose. The measurement levels and the ranges of these variables have been included in Table S1 as well.

#### 2.3.1. Predictors

A wide range of tests and questionnaires were completed at baseline to describe patients’ physical, emotional, and social status. This paragraph gives an overview of the specific umbrella terms used to organize the different variables and the used measurement instruments. Five terms were chosen to provide an overview: (1) generic characteristics; (2) physical factors; (3) psychological factors; (4) social factors; and (5) measures of COPD severity. Table 1 provides an overview of all measurements performed at baseline. The same equipment and standardized methods were used for all examinations. All questionnaires were completed in the presence of a researcher or research assistant to minimize the possibility of missing data. The researcher and the research assistant were not blinded as impact assessor of the study. Handling of missing data was carried out according to the guidelines of Harrell [30]. Missing values in the candidate predictors were imputed by: the mean or the mode if the variable was missing <5%; an estimate from a regression model if the variable was missing 5–15%; or the variable was excluded from the analysis if the variable was missing >15%. The percentages of missing values per predictor and which imputation techniques were used are reported in Online Supplementary Materials Table S2.

#### 2.3.2. Outcome

The key outcome was an eCOPD-related hospitalization during 12 months follow-up. This is a binominal variable categorized as “no hospitalization during follow-up” and “hospitalization during follow-up”.

### 2.4. Statistical Analyses

Data are presented as mean ± standard deviation, frequencies, and median ± interquartile range (IQR), as deemed appropriate. Continuous variables were compared using independent sample t-tests or Mann–Whitney U tests (depending on distribution), while categorical data were compared using Chi-squared or Fisher exact tests. 

A binomial logistic regression was conducted to explore which factors might predict the probability of an eCOPD-related admission. As the final study cohort consisted of 128 participants, it was not possible to include all variables in the model. Therefore, preliminary parameters were identified based on literature and univariate pre-screening before performing the logistic regression. Eighteen variables were significant predictors (*p* ≤ 0.05) for an eCOPD-related admission during follow-up: peripheral capillary oxygen saturation (SpO_2_), forced vital capacity (FVC), inspiratory vital capacity (VC_in_), forced expiratory volume in 1 s (FEV_1_), current smoking status, work situation, timed-up-and-go time (TUG-time), oxygen use, EuroQol 5-dimensions visual analogue scale (EQ-5D VAS score), COPD assessment test (CAT) total score, instrumental activities of daily living scale (IADLS) total score, total number of home adaptations and helping devices, Utrecht coping list (UCL) (most dominant coping style), medical outcome study social support survey (MOSSSS) overall support index, self-developed questionnaire to test patients’ knowledge about COPD and healthy lifestyle (COPD knowledge questionnaire (CIROPD)) percentage correctly answered questions, and eCOPD-related admission(s) prior to baseline. Four different models (physical, psychological, social, and COPD severity) were built as extensions of a baseline model, including: eCOPD-related admission(s) prior to baseline, gender, FVC, and current smoking status. The four models included physical, psychological, social, or COPD severity specific variables, as presented in Table 1.

Per these four models, the most determinant significant factors (*p* ≤ 0.10) were chosen to build the final overall prediction model. The variables were selected based on backward likelihood ratio selection. The significance level of the change in estimate was set at 10%. Multicollinearity was checked by using Pearson correlation coefficients and collinearity diagnostics [variance inflation factor (VIF) and tolerance]. Correlations above 0.7, VIF >10, and tolerance <0.1 indicated a violation of the multicollinearity assumption. Additionally, continuous variables were checked against having a linear relationship with the log of the outcome. All significant interactions (*p* ≤ 0.05) indicated a violation of the linearity of the logit assumption.

Receiver operating characteristic (ROC) analysis was conducted. The area under the ROC curve (AUC) of the different models were reported. Additionally, diagnostic measures such as sensitivity, specificity, positive predictive value (PPV), and negative predictive value (NPV) were reported as well. A priori, a two-sided level of significance was set at *p* ≤ 0.05 for all tests. Data were analyzed using IBM SPSS Statistics for Windows, version 24.0.

## 3. Results

### 3.1. Baseline Characteristics

A total of 194 patients with COPD volunteered to participate. Sixty-six patients were excluded, as they had missing data in relevant outcomes, such as hospitalization during follow-up. The final group of 128 participants was used to perform the statistical analyses. Baseline characteristics of the cohort are described in Table 2. The majority of the participants were above the age of 60 years and male. Only a small number of participants were actively smoking, and most patients had one or more eCOPD-related admission(s) in the 12 months prior to enrollment. During the 12 month follow-up, 31 (24.2%) participants were admitted for eCOPD.

In the bivariate analyses, admission for eCOPD was significantly more frequent in participants with: lower SpO_2_ values at baseline, poorer CAT scores, lower FEV_1_, and a history of eCOPD-related admission(s) prior to baseline. Additionally, eCOPD-related admissions were also more frequent in patients who: had more home adaptations, used more helping devices, had lower IADLS score, had a score <60% correct of the CIROPD questionnaire and a significantly lower score on the EQ-5D VAS scale. No differences were found between patients hospitalized for eCOPD and patients not hospitalized for eCOPD for other baseline characteristics.

### 3.2. Predictors of eCOPD-Related Hospitalization

Please see online Supplementary Materials Table S3 through Table S6 for the details of physical, psychological, social, and COPD severity model. In the final overall prediction model, current smoking status, FVC, TUG-time, CIROPD_%correct_ answered questions, eCOPD-related admission(s) prior to baseline, gender, CDS total score, and MOSSSS overall support index were entered as independent variables. After stepwise exclusion, six predictors were selected to build the final prediction model, as shown in Table 3. The final model containing these predictors was statistically significant (χ^2^_(df = 7, *n* = 128)_ = 43.84, *p* = 0.00), suggesting that the model was able to distinguish between patients hospitalized due to eCOPD and patients who were not hospitalized. The Hosmer–Lemeshow Goodness-of-Fit Test was 3.39 with a significance level of 0.91, suggesting a well calibrated model in the developmental sample. The model as a whole explained between 29.0% (Cox and Snell R square) and 43.3% (Nagelkerke R squared) of the variance in an eCOPD-related hospitalization and correctly classified 79.7% of the cases.

As shown in Table 3, all six variables made a unique statistically significant contribution to the model. The strongest predictor of an eCOPD-related hospitalization was TUG-time, presenting an odds ratio of 14.2, suggesting that patients who had a TUG-time ≥14 s have 14.2 times the odds of being hospitalized due to eCOPD compared to patients who had a TUG-time <14 s when controlling for all other factors in the model.

As an example, if a clinically independent and stable patient currently does not smoke, has a FVC (%pred) of 90%, has a TUG-time of 8.9 s, correctly answered 83% of the questions of the CIROPD questionnaire, does not have a history of eCOPD-related hospitalization(s), and is independent of care from others (CDS total score of 72 points), then this patient has a probability of being hospitalized for eCOPD during 1 year follow-up of 11.2%. However, when this same patient has a TUG-time of 14.6 s instead of 8.9 s, then the probability of being hospitalized for eCOPD during 1 year follow-up is 64.1%. This indicates that TUG-time is a very strong predictor in this model.

The odds ratio of CIROPD_%correct_ (60%–75%) was less than one, suggesting that patients who scored between 60%–75% correct on the CIROPD questionnaire have 0.30 times the odds of being hospitalized for eCOPD compared to patients who scored <60% correct on the CIROPD questionnaire when controlling for other factors in the model. This means that a higher score for the CIROPD questionnaire (that is, more knowledge about their disease and healthy lifestyle) could help prevent an eCOPD-related admission.

Furthermore, patients who currently smoke, have a history of eCOPD-related admission(s) prior to baseline, and had higher scores on the CDS questionnaire and thus are less care-dependent are more likely to be hospitalized than patients who do not smoke (anymore), who do not have a history of eCOPD-related admission(s), and who had lower scores on the CDS questionnaires and are more care-dependent, respectively. FVC (%pred) appeared to be a protective factor for an eCOPD-related admission during follow-up. However, the odds ratio was close to one, suggesting that this is not a strong predicting factor.

### 3.3. Diagnostics

The final overall prediction model had a sensitivity of 61.9%, a specificity of 83.2%, a PPV of 41.9%, and a NPV of 91.8%. This suggests that this model more accurately predicts which patients will not get hospitalized compared to the patients who will get hospitalized. The ROC curve had an area of 86.1% (95% CI: 79.4%–92.7%), suggesting a good accuracy of the overall prediction model.

## 4. Discussion

In this study, we prospectively assessed the frequency of and the risk factors for an eCOPD-related hospitalization. One out of four patients with COPD were admitted to the hospital at least once during 1 year follow up. A high TUG-time (≥14 s), eCOPD-related admission(s) prior to baseline, currently active smoking status, lower FVC (%pred), higher CDS total score, and lower CIROPD_%correct_ are statistically significant predictors of an eCOPD-related hospital admission.

### 4.1. Predictors of eCOPD-Related Hospital Admission

In our study, TUG-time was the strongest risk factor of future eCOPD-related admissions compared to the other predictors. The TUG test can be seen as an exercise field test to assess physical functioning in patients with COPD [53,54]. It is expected that patients with high risk of falling (TUG ≥14 s) have an increased risk of being admitted to the hospital. This is in line with previous findings that patients with a lower walking distance on the 6 min walk test, another field-based exercise test, have an increased risk for an eCOPD-related hospital admission compared to patients with higher walking distance on the 6 min walk test [55].

CDS total scores represent the patient’s degree of care dependency. Patients with a low total score of the CDS suggest that they are highly dependent on care from others. It is expected that those patients are more likely to be hospitalized compared to patients who function well independently. Previously, Janssen and colleagues showed that patients with advanced chronic organ failure (including COPD) with low CDS scores were at increased risk of dying [26]. Our study found that patients with low total scores of the CDS do have lower odds of being prospectively hospitalized for eCOPD compared to patents with high total scores of the CDS. Our finding therefore contradicts the previous findings by Janssen and colleagues [26]. This could be explained by the fact that only a very small percentage (12%) of the people who participated in the Home Sweet Home study had a low total score of the CDS (<60 points), indicating the patients with a care dependency degree of “completely dependent” to “partly dependent.” Therefore, care dependent patients were underrepresented when building this model. Furthermore, all participants were living together with their partner and/or other relatives. Therefore, it is likely that informal care occurred. The CDS total scores might be biased by the occurrence of informal care.

Additionally, eCOPD-related admission(s) prior to baseline was another strong predictor in our study. This finding is in line with previous findings [18,56]. Besides that, a history of eCOPD-related admission(s) is associated with an increased likelihood of readmission; it is also associated with an increased risk of death [7]. An explanation of this strong predictive value could be that participants who have previously been hospitalized for eCOPD are at a more severe stage of the disease compared to participants who were not previously hospitalized. This could be confirmed by decreased pulmonary functioning, lower SpO_2_ values, increased CAT-scores, and overall worse health status.

Smoking history was found as a risk factor by our study. However, the review by Halpin and colleagues (2017) found mixed evidence regarding the impact of smoking status on the risk of an eCOPD-related admission [56]. The influence of having formerly smoked therefore remains ambivalent.

CIROPD_%correct_ is a protective factor of future eCOPD-related admissions. CIROPD is a questionnaire that tests the knowledge of patients about COPD and healthy lifestyle. This suggests that people who have a high percentage of correctly answered questions have more knowledge about their disease and healthy lifestyle compared to people who have a low percentage of correctly answered questions. Therefore, you can assume that people with more knowledge about their disease recognize when they need to seek help earlier than those who lack such knowledge. These findings again emphasize the importance of education for patients with COPD [57,58].

Lastly, FVC at baseline was a predictor of future eCOPD-related admissions. Pulmonary function tests are proven to be significant predictors of future eCOPD-related admissions [15,16,17]. Nevertheless, it is expected that FEV_1_ has a bigger impact compared to the FVC. However, in our study, it was the other way around. The FEV_1_ had a non-significant association with eCOPD in the model. Therefore, the FEV_1_ was excluded from the model(s).

Interestingly, the identified factors are all (at least to some extent) possible treatment targets. Indeed, education may increase patients’ knowledge, exercise can reduce TUG-time, pulmonary rehabilitation can reduce the degree of care dependency, and smoking cessation can reduce the number of active smokers [54,59].

### 4.2. Methodological Considerations

Our study has several strengths and limitations. Pre-selection of the final factors was based on univariate analyses and literature suggestions. Pre-selection using univariate analysis is not a methodologically sound way of performing a binominal logistic regression, as this includes selection bias. However, this was necessary because of the limited number of events.

Additionally, our study was mostly an explorative study based on which no reliable model can be made to predict future eCOPD-related admissions. The cohort was used to develop an explorative model. The sample size was too small to split into a developmental and a validation cohort. Therefore, we were unable to externally validate the different models. Another way to externally validate the model is to use other available COPD cohort studies, such as the ARCTIC observational cohort study [60], the Hokkaido COPD cohort study [61], or SPIROMICS (Subpopulations and Intermediate Outcomes in COPD Study) [62]; however, this was not the aim of this study. Obviously, the current results need to be confirmed by others. Future studies could perform external validation in the cohorts listed to corroborate the current findings.

This study used data from a prospective, longitudinal observational study susceptible to three main types of biases: selection bias, information bias, and confounding [63,64]. The most pertinent consequence of selection bias in this context is the differential losses to follow-up. The biggest concern for information bias is that the outcome is reported requiring involvement of an observer and/or responder. Additionally, this study made use of many different questionnaires, which were subjected to recall bias. Finally, when examining the association between the risk factors and an eCOPD-related admission, the association could be influenced by other variables associated with independent or dependent variables, thus called confounding. Confounding was taken into account as far as possible; however, a small risk of missing out confounders remained.

## 5. Conclusions

In summary, this prospective observational study of a small population of participants with COPD GOLD-class II, III, or IV showed that, among a wide range of potential risk factors, only high TUG-time (≥14 s), eCOPD-related admission(s) prior to baseline, currently smoking, lower FVC (%pred), higher CDS total score, and lower percentage of correctly answered questions of the CIROPD questionnaire were independently associated with an increased risk of future eCOPD-related admissions. In this study, no other physical, psychological, and/or social factors were found to be associated with an eCOPD-related admission during follow-up. Future studies should explore other variables regarding physical, psychological, and social factors regarding the prediction of an eCOPD-related admission. Additionally, a bigger sample size is warranted to investigate if these results are sustainable in larger sample sizes and in different healthcare systems.

## Figures and Tables

**Table 1:** Overview of all measurements performed at baseline.

Category	Measurement Instrument
**Generic characteristics**	
**Background information**	Age; Gender; Marital status; Working status; Educational background; Monthly income
**Smoking history and habits**	Self-developed questionnaire
	Smoking history
	Fagerström test for nicotine dependence [31]
**Clinical characteristics**	Height; Weight; Body Mass Index (BMI)
	Post-bronchodilator spirometry [32]: Forced Vital Capacity (FVC), Forced Expiratory Volume in 1 sec. (FEV_1_), Inspiratory Vital Capacity (VC_in_), and Peak Expiratory Flow (PEF)
	Resting: Blood pressure; Heart rate (HR); Peripheral capillary oxygen saturation (SpO_2_)
	Oxygen use, Non-invasive ventilation (NIV) use
**Medical history**	Charlson comorbidity index [33]
	Current medication
	Participated in pulmonary rehabilitation program
	Hospitalization prior to baseline; Time between previous admission and baseline
	Home adaptations and aids
**Physical Factors**	
**Daily functioning**	Canadian Occupational Performance Measure (COPM) [34]
	Instrumental Activities of Daily Living Scale (IADLS) [35]
**Symptoms of fatigue**	Subscale of the Checklist Individual Strength (CIS) (subjective) [36]
**Mobility**	Timed-Up-and-Go test (TUG) [37]
**Psychological Factors**	
**Symptoms of Anxiety and depression**	Hospital Anxiety and Depression Scale (HADS) [38]
**Chronic obstructive pulmonary disease (COPD) specific knowledge**	COPD knowledge questionnaire (CIROPD)
**Coping**	Utrecht Coping List (UCL) [39]
**Social Factors**	
**General well-being**	Assessment of Quality of Life with 8 dimensions (AQoL-8D) [40]
	Care Dependency Scale (CDS) [41]
**Physical activity and motivation**	Behavioral Regulation in Exercise Questionnaire (BREQ-2) [42]; Social-individual focus
**Social support**	Medical Outcome Study Social Support Survey (MOSSSS) [43,44]
**Quality of the relationship**	Dutch relationship questionnaire (NRV) [45,46]
**Measures of COPD severity**	
**Disease-specific health status**	COPD Assessment Test (CAT) [47]; Symptoms Visual Analogue Scale (VAS) [48]; Symptoms (dyspnea and fatigue)
**Generic health status**	12-item short form health survey (SF-12) [49,50]
	EuroQol 5-dimensions (EQ-5D) [51]
**Dyspnea**	Modified Medical Research Council (mMRC) [52]

**Table 2:** Baseline demographic and clinical characteristics of study population.

	Total Cohort	Hospitalization During Follow-Up	No Hospitalization During Follow-Up	*p*-Value
*n* (male/female)	128 (73/55)	31 (15/16)	97 (58/39)	0.26
Age (years)	65.5 ± 8.2	65.2 ± 8.2	65.6 ± 8.2	0.85
BMI (kg/m^2^)	25.4 (22.6–29.1)	24.7 (22.1–25.9)	25.8 (22.9–29.4)	0.06
SpO2 (%)	95 (93–96)	93 (90–96)	95 (93–97)	0.01 *
HR (bpm)	77 ± 13	79 ± 15	76 ± 12	0.39
Long-term oxygen use (yes)	29 (22.7%)	11 (35.5%)	18 (18.6%)	0.08
NIV (yes)	8 (6.3%)	0 (0%)	8 (8.2%)	0.20
CCI (points)	2 (1–3)	2 (1–3)	2 (1–3)	0.99
Current smoker (yes)	19 (14.8%)	8 (25.8%)	11 (11.3%)	0.08
CAT total score (points)	21.0 ± 6.6	23.7 ± 5.5	20.2 ± 6.7	0.01 *
**History of eCOPD**				0.00 *
No	59 (46.1%)	5 (16.1%)	54 (55.7%)	
Yes	69 (53.9%)	26 (83.9%)	43 (44.3%)	
**mMRC dyspnoea grade**				0.64
0–1	35 (27.3%)	7 (22.6%)	28 (28.9%)	
2–4	93 (72.7%)	24 (77.4%)	69 (71.1%)	
**Lung function**				
FEV_1_ (%pred)	48.5 (33.0–61.8)	39 (27.0–56.0)	50 (34.5–63.5)	0.02 *
FVC (%pred)	96.8 ± 19.8	88.4 ± 20.3	99.5 ± 18.9	0.01 *
**Coping style**				0.01 *
Actively addressing	60 (46.9%)	15 (48.4%)	45 (46.4%)	
Palliative response	26 (20.3%)	9 (29.0%)	17 (17.5%)	
Avoid	38 (29.7%)	4 (12.9%)	34 (35.1%)	
Passive reaction	4 (3.1%)	3 (9.7%)	1 (1.0%)	
Looking for social support	0 (0%)	0 (0%)	0 (0%)	
Emotional expression	0 (0%)	0 (0%)	0 (0%)	
Comforting thoughts	0 (0%)	0 (0%)	0 (0%)	

Abbreviations: N = number, BMI = body mass index, SpO_2_ = peripheral capillary oxygen saturation, HR = heart rate, NIV = non-invasive ventilation, CCI = Charlson Comorbidity Index, CAT = COPD Assessment Test, eCOPD = exacerbation of COPD, mMRC = Modified Medical Research Council Dyspnoea Scale, FEV_1_ = forced expiratory volume in 1 s, FVC = forced vital capacity.* *p* ≤ 0.05.

**Table 3:** Logistic regression predicting the probability of eCOPD-related admission—overall prediction model.

Variable	B	S.E.	Wald	df	*p*-Value	OR	95% CI for OR	
							Lower	Upper
Current smoking status, Smoker	1.37	0.68	4.01	1	0.04	3.93	1.03	14.98
FVC (%pred)	−0.04	0.01	6.35	1	0.01	0.97	0.94	0.99
TUG-time, ≥14 s	2.65	1.22	4.74	1	0.03	14.16	1.30	153.79
CIROPD_%correct_, <60%			6.70	2	0.03			
CIROPD_%correct_, 60%–75%	−1.21	0.66	3.34	1	0.07	0.30	0.08	1.09
CIROPD_%correct_, >75%	0.66	0.64	1.07	1	0.30	1.94	0.55	6.78
Previously hospitalized, Yes	2.30	0.65	12.37	1	0.00	9.98	2.77	35.98
CDS total score	0.11	0.05	5.22	1	0.02	1.12	1.02	1.23
Constant	−7.05	3.36	4.41	1	0.04	0.00		

Abbreviations: B = regression coefficient, S.E. = standard error, df = degrees of freedom, OR = odds ratio, CI = confidence interval, FVC = forced vital capacity, TUG-time = timed-up-and-go test, CIROPD = CIRO developed questionnaire to test patient knowledge of COPD and healthy lifestyle, CDS = care dependency scale.

## Supplementary Materials

The following are available online at https://www.mdpi.com/2077-0383/9/3/636/s1, Table S1: Additional information measurement instruments, Table S2: Imputation characteristics, Table S3: Physical model, Table S4: Psychological model, Table S5: Social model, Table S6: COPD severity model.
