# Supplementary material for: Physical, Psychological, and Social Factors Associated with Exacerbation-Related Hospitalization in Patients with COPD
Source: J Clin Med. 2020 Feb 27;9(3):636. doi: 10.3390/jcm9030636 (PMC7141103; doi:10.3390/jcm9030636)
Supplement: Supplementary file 1 [file jcm-09-00636-s001.pdf]

Online Supplementary Materials

Table S1. Additional information measurement instruments

Category	Variable	Range as measured in study population	Measurement level
Daily functioning	Canadian Occupational Performance Measure (COPM): Performance and Satisfaction [34]	Range from 1 (not at all able/satisfied) – 10 (able to perform extremely well/extremely satisfied)	Ratio scale
	Instrumental Activities of Daily Living Scale (IADLS)[35]	Range from 0 (independent) – 8 (dependent)	Ratio scale
Disease-specific health status	COPD Assessment Test (CAT) [47]	Range from 0 (no problems) – 40 (worst)	Ratio scale
	Symptoms (dyspnoea and fatigue)	Range from 0 (no problems) – 10 (worst)	Ratio-scale
	Symptoms VAS	Range from 0 (no symptom) – 100mm (worst) (total number of symptoms; VAS score >30)	Ratio-scale
Generic health status	12-item short form health survey (SF-12)[49, 50]	Two subscales: Physical & Mental<br>A score higher or lower than 50 means that you score higher or respectively lower than the general population (SD = 10).	Ordinal
	EuroQol 5-dimensions (EQ-5D) AND EQ-5D VAS [51]	Total score measured by using algorithm (See user manual); EQ-5D VAS (range from 0 (worst) – 100mm (perfect health))	Ratio-scale
Dyspnoea	modified Medical Research Council (mMRC) dyspnoea scale [52]	Range from 0 (no dyspnea) – 4 (extreme dyspnea)	Ratio scale
Symptoms of fatigue	Subjective fatigue subscale of the Checklist Individual Strength (CIS) [36]	8 items score options between 1 – 7 (summarized score); Range from 8 (best) – 56 (more severe fatigue)	Ratio-scale
Symptoms of anxiety and depression	Hospital Anxiety and Depression scale (HADS) [38]	Anxiety and Depression separated<br>Range from 0 (no anxiety or depression) – 21 (anxiety or depression)	Ratio-scale
General well-being	Assessment of Quality of Life with 8 dimensions (AQoL-8D) [40]	Utility (measured 8 dimensions).	Ratio-scale
	Care Dependency Scale (CDS)[41]	15 items score options between 1 – 5; added up > range from 15 (dependent) – 75 (independent)	Ratio-scale
Mobility	Time-Up-and-Go test [37]	<14 sec. (low risk for falling) vs. ≥14 sec.(high-risk for falling)	Ratio-scale
COPD specific knowledge	CIROPD knowledge questionnaire	Percentage correctly answered questions → pessimistic scenario. (Correctly answered questions / total number of questions).<br>Ranges from 0% (no correctly answered questions) – 100% (only correctly answered questions)	Ratio-scale

Coping	Utrecht Coping List (UCL) [39]	Most dominant copying style: Actively addressing, palliative response, avoid, searching social support, passive reaction, emotional expression, comforting thoughts	Nominal-scale
Physical Activity and motivation	Behavioural Regulation in Exercise Questionnaire (BREQ-2) [42]	RAI (=relative autonomy index) > Degree of self-determinacy	Ratio-scale
	Social-individual focus	8 questions (range from 1-7) → total scores range from 8 (individually) – 56 (companion).	Ratio-scale
Smoking status	Self-developed questionnaire	Qualitative	Qualitative
	Fagerström test for nicotine dependency[31]	Range 0 (nicotine independent) – 10 (nicotine dependent)	Ratio-scale
	Current smoking status	Currently smoking vs. non-smoker	Binominal
Social support	Medical Outcome Study Social Support Survey (MOSSSS) [43, 44]	Overall support index (range from 1 (no social support) – 15 (social support))	Ratio-scale
Quality of the relationship	Dutch relationship questionnaire (NRV) [45, 46]	True/False questions (range 0 (worse) – 80 (high relationship quality))	Ratio-scale
Clinical characteristics	Body Mass Index	BMI in kg/m^2	Ratio-scale
	Height	Height in cm	Ratio-scale
	Post bronchodilator spirometry [32]	% improvement in FEV_1, FVC, VCin, and PEF	Ratio-scale
	Resting blood pressure	BP in mmHg	Ratio-scale
	Resting heart rate	HR in bpm	Ratio-scale
	Oxygen use	No vs. Yes	Binominal
	Non-invasive mechanical ventilation (NIMV)	No vs. Yes	Binominal
	Resting peripheral capillary oxygen saturation	SpO_2 in %	Ratio-scale
Demographics	Age	Can range from 0 - ∞ (0 – 100 is more realistic)	Ratio-scale
	Gender	Male vs. Female	Nominal-scale
	Marital status	Married vs. Unmarried	Binominal
	Working status	Entrepreneur (>=12hours a week), retired, domestic work, student, volunteer, (long-term) incapacitated to work, unemployed (or <12 hours a week)	Nominal-scale
	Educational Background	None, primary education, secondary education, and higher education/university	Ordinal-scale
	Monthly income	Less than €1470,- gross per month, between €1470,- and €2546,- gross per month, more than €2546,- gross per month	Ordinal-scale
Medical history	Charlson comorbid index [33]	Range 0 (low likelihood of dying) – 40 (increased likelihood of dying)	Ratio-scale
	Current medication	Total number of used medication	Ratio-scale
	Participated in rehabilitation program	No; Yes, pulmonary rehabilitation; Yes, other rehabilitation	Nominal-scale
	Hospitalization for eCOPD before index admission	No hospitalization before baseline, and hospitalization before baseline.	Ordinal-scale
	Home adaptations and aids	Total number of home adaptations and aids	Ratio-scale

Table S2. Imputation characteristics

Variable name	Missing rate (%)	Imputed	Imputed with	Assumption
Age	0.0	N/A		
Gender	0.0	N/A		
Height	0.0	N/A		
BMI	0.0	N/A		
SpO_2	0.0	N/A		
HR	0.0	N/A		
BP_Systolic	0.0	N/A		
BP_Diastolic	0.0	N/A		
VC_{in}_pred	0.0	N/A		
FVC_pred	0.0	N/A		
FEV_1_pred	0.0	N/A		
PEF_pred	0.0	N/A		
mMRC	0.0	N/A		
Total_number_medications	0.0	N/A		
RE_SMOKE_status	0.0	N/A		
RE_Fagerstrom_CAT	1.6	0	Mode	MCAR
Packyears	0.0	N/A		
RE_Marital_status	0.0	N/A		
Educational_background	0.0	N/A		
Work_situation	0.0	N/A		
Monthly_income	1.6	1	Mode	MCAR
Totalscore_Charlson	0.0	N/A		
RE_Revalidationprogram	0.0	N/A		
TUG	0.8	10.21	Mean	MCAR
SMOKE_timestopped	16.4		Excluded for high missingness	
SMOKE_stimulation LO	16.4		Excluded for high missingness	
SMOKE_what	85.2		Excluded for high missingness	
SMOKE_timestarted	85.2		Excluded for high missingness	
O_2_daily	85.9		Excluded for high missingness	
O_2_start	77.3		Excluded for high missingness	
SMOKE_residents	0.0	N/A		
SMOKE_in_your_presence	0.0	N/A		
O_2	0.0	N/A		
NIMV	3.1	0	Mode	MCAR
COPM_Totalscore_performance	0.8	4.75	Median	MCAR
COPM_Totalscore_satisfaction	0.8	4	Median	MCAR
Sympt_1 (dyspnoea)	0.0	N/A		
Sympt_2 (fatigue)	0.0	N/A		

Symptoms_VAS	0.0	N/A		
Self_Efficacy_Totalscore	0.0	N/A		
EQ5D_Dutch	0.0	N/A		
EQ5D_VAS	0.0	N/A		
CAT_totalscore	0.8	21	Median	MCAR
IADLS_totalscore	0.0	N/A		
Adaptations_Home_total	0.0	N/A		
Helping_Devices_total	0.0	N/A		
SF12physical	0.0	N/A		
SF12mental	0.0	N/A		
CDS_totalscore	0.0	N/A		
CIS_totalscore	0.0	N/A		
UCL_Dominant_copingstyle	0.0	N/A		
Social_Individual_Focus_Total	0.0	N/A		
AQoL8DUtility	0.0	N/A		
CIROPD_right	0.0	N/A		
CIROPD_wrong	0.0	N/A		
CIROPD_dontknow	0.0	N/A		
MOSSSS_Overall_support_index	2.3	4.22	Mean	MCAR
BREQ2_RAI	0.0	N/A		
HADS_A	0.0	N/A		
HADS_D	0.0	N/A		
NRV_totalscore	9.4	Average of 5 imputations	Multiple imputation (regression method)	MCAR
HOSP_before	0.0	N/A		
HOSP_FU	0.0	N/A		

Abbreviation: N/A = Not Applicable, MCAR = missing completely at random.

Table S3. Physical model

Variable	B	S.E.	Wald	df	P-value	Odds Ratio	95% CI	
							Lower	Upper
Previously hospitalized, Yes	1.70	0.57	8.82	1	0.00	5.46	1.78	16.71
FVC (%pred)	-0.02	0.01	3.89	1	0.05	0.98	0.95	1.00
Current smoking status, Smoker	1.43	0.61	5.50	1	0.02	4.17	1.27	13.76
TUG-time, ≥14sec	1.74	0.90	3.70	1	0.05	5.69	0.97	33.44
Constant	0.02	1.25	0.08	1	0.78	0.70		

Abbreviations: B = regression coefficient, S.E. = Standard Error, df = degrees of freedom, CI = confidence interval, FVC = Forced Vital Capacity, TUG = Timed-Up-and-Go test.

Table S4. Psychological model

Variable	B	S.E.	Wald	df	P-value	Odds Ratio	95% CI	
							Lower	Upper
Gender, Female	1.01	0.53	3.67	1	0.05	2.75	0.98	7.72
Previously hospitalized, Yes	1.92	0.58	10.93	1	0.00	6.85	2.19	21.44
FVC (%pred)	-0.03	0.01	5.34	1	0.02	0.97	0.95	0.99
Current smoking status, Smoker	1.16	0.67	2.98	1	0.08	3.19	0.85	11.91
$CIROPD_{\%correct}$, <60%	REF	REF	8.02	2	0.02			
$CIROPD_{\%correct}$, 60%–75%	-1.54	0.62	6.09	1	0.01	0.21	0.06	0.73
$CIROPD_{\%correct}$, >75%	0.25	0.60	0.18	1	0.68	1.28	0.40	4.17
Constant	0.18	1.29	0.02	1	0.89	1.19		

Abbreviations: B = regression coefficient, S.E. = Standard Error, df = degrees of freedom, CI = confidence interval, FVC = Forced Vital Capacity, CIROPD = CIRO developed questionnaire to test patient's knowledge of COPD and healthy lifestyle.

Table S5. Social model

Variable	B	S.E.	Wald	df	P-value	Odds Ratio	95% CI	
							Lower	Upper
Previously hospitalized, Yes	2.31	0.63	13.66	1	0.00	10.10	2.96	34.45
FVC (%pred)	-0.04	0.01	6.63	1	0.01	0.96	0.94	0.99
Current smoking status, Smoker	1.29	0.62	4.37	1	0.04	3.63	1.08	12.15
CDS total score	0.05	0.03	3.04	1	0.08	1.05	0.99	1.12
MOSSSS: overall support index	-0.52	0.29	3.30	1	0.07	0.59	0.34	1.04
Constant	-0.90	2.21	0.17	1	0.68	0.41		

Abbreviations: B = regression coefficient, S.E. = Standard Error, df = degrees of freedom, CI = confidence interval, FVC = Forced Vital Capacity, CDS = care dependency scale, MOSSSS = Medical Outcomes Study Social Support Survey.

Table S6. COPD severity model

Variable	B	S.E.	Wald	df	P-value	Odds Ratio	95% CI	
							Lower	Upper
Gender, Female	0.84	0.50	2.85	1	0.09	2.32	0.87	6.16
Previously hospitalized, Yes	1.94	0.56	11.82	1	0.00	6.97	2.30	21.10
FVC (%pred)	-0.03	0.01	6.27	1	0.01	0.97	0.94	0.99
Current smoking status, Smoker	1.30	0.61	4.51	1	0.03	3.67	1.11	12.17
Constant	0.02	1.25	0.00	1	0.98	1.02		

Abbreviations: B = regression coefficient, S.E. = Standard Error, df = degrees of freedom, CI = confidence interval, FVC = Forced Vital Capacity.
